# Medical students’ experience with accessing medical records in Saudi Arabia: a descriptive study

**DOI:** 10.1186/s12909-021-02715-7

**Published:** 2021-05-12

**Authors:** Jwaher A. Almulhem

**Affiliations:** Medical Informatics and E-learning Unit, Medical Education Department, College of Medicine, King Saud University, Riyadh, Kingdom of Saudi Arabia

**Keywords:** Medical students, Access to medical record, Saudi Arabia, Access to electronic health record, Medical education

## Abstract

**Background:**

Medical students can enhance their knowledge by accessing patients’ medical records and documenting patient care. This study assessed medical students’ access to paper medical records and electronic health records (EHRs) in Saudi Arabia and compared students’ experience of accessing paper medical records and EHR from their perspective.

**Methods:**

This cross-sectional study enrolled second-year to intern medical students randomly from different medical colleges in Saudi Arabia. A self-developed survey was administered to them. It comprised 28 items in three sections: general information about medical students and their level of accessing medical records, their experience with the medical record system used in hospitals, and their preference for the medical record type.

**Results:**

62.8% of participants had access to medical records, with 66.1% of them having access to EHRs and 83.27% had read-only access. The EHR group and paper group mostly liked being able to reach medical records effortlessly (70.1% and 67.1%, respectively). The EHR group had a better experience compared to the paper group with *U* = 5200, Mean Rank = 122.73, *P* = .04. Students who trained in University – owned and National Guard hospitals had better experiences compared to students who trained in other hospitals with Mean Ranks =122.35, and 147.99, respectively.

**Conclusion:**

Incorporating EHR access into the medical curriculum is essential for creating new educational opportunities that are not otherwise available to medical students.

**Supplementary Information:**

The online version contains supplementary material available at 10.1186/s12909-021-02715-7.

## Background

Medical students can improve their knowledge by accessing patients’ medical records and documenting patient care [1]. With regard to learning theory, medical students’ participation in documenting patient care is an educational activity [2]. Acquiring documentation skills changes depending on the number of years in medical schools, starting with recording clinical data in preclinical years, followed by ranking, combining, and incorporating clinical information during clinical years. Finally, a medical student’s documentation becomes a method of information exchange and communication between different health providers [3].

It is critical for medical students to have access to medical records for educational purposes. When healthcare organizations use traditional paper medical records, students did not have any barrier with accessing medical records [4] and have more opportunity in entering patients’ orders [5]. They usually gain required skills of writing notes and entering orders as part of their clinical experience. However, transition to electronic health record (EHR) necessitate acquiring essential EHR-related competencies after graduation [6]. Medical students need to learn about several aspects of using EHRs, including recording patients’ medical histories and physical examination; documenting and ordering laboratory tests, radiology, medication, and consultation care; and understanding the method of using EHRs in specific hospitals [7, 8]. Third-year medical students spend more than 4 h using EHRs and write, on average, three notes in medical records per day [9].

Increasing learning experience and familiarity with EHR among future physicians, will enhance positive impact resulted from using such system. Starting with implementation phased, McGinn et al. [10] indicated familiarity and ability of using EHR is one of the facilitator factors of implementation process. Physicians who had 4 years or more experience with EHR were more likely to agree about the positive influence related to EHR use including improved patient care, data confidentiality, and reduced costs [11]. Familiarity with EHR also helps health care providers to deal with safety and maintenance issues such as hardware and software failures, wrong patient identification, and subversion of clinical decision support protocols [12]. In addition, exposure to different EHR systems implemented in hospitals will enhance medical students’ experience of training and use of these systems with different features and user interfaces [13].

Duke, Frankel, and Reis [14] reported that providing full medical record access to medical students is important as it will help them understand how to extract and retrieve patients’ medical histories and other significant information. Specifically, medical students need to use clinical decision support systems (CDSS) and computerized physician order entries so that they can use these systems when providing medical care in the future. Biagioli et al. [15] linked a lack of proper EHR training to skill deficiencies in several EHR-related core aspects such as medical history review, medication reconciliation, and allergy reconciliation. In addition, most medical students’ EHR skills do not improve as the year proceeds, if they do not acquire these skills in their early undergraduate medical years. Providing medical students access to EHRs will help them track patients and record medical procedures, improve self-directed learning, and increase their understanding of diagnostic and prognostic consequences [16, 17].

Hammoud et al. [18] conducted a national survey of clerkship directors to explore the current practice of the use of EHRs among US medical students. They found that only 64% of surveyed programs allow medical students to use EHRs, with two-thirds of them allowed to view EHRs, write notes, and enter orders. The Liaison Committee on Medical Education (LCME) reported that the level of access differs across different types of hospitals. In addition, ~ 30% of University-owned hospitals allow medical students to view EHRs without entering or modifying patient information [6, 19]. The percentage of medical students who access EHRs has increased from 78 to 93%, while the mean percentage of clerkships in which a medical student uses paper medical records has decreased over time [20].

With transferring from paper medical record to EHR [6], hospitals restrict medical students access to EHRs for several reasons. In the LCME Annual Medical School Questionnaire Part II, 45 of 140 hospitals indicated that the main reason for preventing medical students from entering information into the EHR system is hospital and/or medical staff requirements and another reason is the EHR system structure [6]. Financial aspects related to providing medical students with computers, EHR licenses, and authorization cardinals to access various systems also play a role in access restriction [18]. In addition, hospitals might implement policies that restrict documentation by medical students in order to avoid regulatory issues, such as Joint Commission citations for use of incorrect abbreviations [3].

The Saudi Medical Education Directives (SaudiMED) requires several learning outcomes and competencies that are anticipated by medical students after their graduation. The framework specified two program learning objectives (PLOs) that related to accessing patient records; communicate with health professionals and patients effectively and use medical informatics systems appropriately during providing healthcare. They elaborate on these PLOs with specific enabling competencies that should be met by medical students before beginning their internship program. Effective communication involves using verbal and documenting skills to disseminate medical information. Using the medical informatics system appropriately through storing, retrieving information, and using this information ethically in providing patient care and health promotion [21].

The Ministry of Health (MOH) launched an E-Health strategy that depends on several dimensions such as patient-centric care and interoperable EHR [22]. A recent study found the attitude towards E-health use was positive among medical students [23]. Indeed, authentic of clinical experiences, which included documenting in patients’ records, was mentioned as a factor for improving Saudi medical students learning environment [24]. MOH stated that “By 2020: 70 % of the population will have digitized unified health records” [25]. A recent study found that most of the medical students favored a reasonable amount of technology in their education. They concluded that the cooperation of information technology (IT) with the curriculum is required to increase the utilization of available resources [26]. Consequently, involving EHR competences in medical students’ curriculum is one of the essential steps to prepare future medical professionals in Saudi Arabia.

Yet, this is the first study in Saudi Arabia to discuss this topic among medical students and understand the educational impact of accessing medical records among medical students from several medical universities and different clinical sittings. Indeed, prior studies focus on impact of medical record in one specialty or individual clinical sitting [27, 28]. Recognizing actual students’ viewpoint regrading accessing medical record and their educational impact have not been extensively reported from students since previous studies depend on medical educators’ point of view [18, 29]. Furthermore, comparing learning experience between students who access traditional paper record and EHR is not clear yet and have not extensively studied in the literature [5]. Accordingly, this study assessed medical students’ access to paper medical records and EHR in Saudi Arabia and compared the experience of accessing paper medical records and EHR from the medical students’ perspective.

Improving understanding of medical record’s educational benefits will encourage medical educators to involve already implemented IT systems in healthcare organizations to enhance the medical education environment since using EHR is compulsory during these days. Recognizing barriers that prevent medical students access to medical record, particularly after shift from paper medical records to EHR, will help academic institutions to be aware of students’ current educational environment and recommend possible solutions.

## Methods

### Study design and the sample

This descriptive, cross-sectional study was conducted with second-year to intern medical students. The number of medical colleges is approximately 31 colleges in Saudi Arabia [30]. Before the communication process, We randomly selected 10 universities and sent a formal letter from the postgraduate and scientific research deanship in King Saud University to the postgraduate and scientific research deanship of these universities. The purpose of these letters is to introduce the researcher and the purpose of this study. Also, it involved asking for permission and facilitation of data collection process among medical students. Only 4 of 10 universities responded.

The study was approved by the King Saud University ethical committee (KSU-KSU-HE-19-374).

### Instrument development and distribution

A self-developed survey was designed after reviewing several studies on medical students’ access to medical records [6, 19, 28, 31]. The survey was developed using the Google Form Survey development tool. Several experts in health informatics, medical education and former medical students reviewed the survey before distribution. According to their suggestions, sequence of the questions and few wording issues of the first draft were identified and corrected before distribution.

Next, the survey was distributed through the medical colleges’ official email, learning management systems, and other formal student groups. To increase the response rate, a second reminder over the medical students’ official email was sent. The data collection started in 3/3/2019 and lasted for 12 months. The required sample size was 384, calculated on the basis of the Kotrlik and Higgins formula [32]. The email also included the study's objectives and a link to participate voluntarily and anonymously. Finally, 388 medical students participated in the study.

The survey comprised 28 items in three sections (see the Additional file 1): (i) general information about the medical students and their level of accessing medical records, (ii) their experience with the medical record system used in hospitals, and (iii) their preference regarding the medical record type they wanted to use in their future practice. Section 1 asked about the medical students’ age, gender, studying year, hospital type, access to medical records (yes/no), access method (free to access, access from IT team, access through a senior’s account, other), type of medical record (paper medical record, EHR), and level of access (read-only, full access). (Having full access means being able to read, review, and enter order/data in the medical record.) To understand the reason behind medical student access restriction, we asked those who did not have access to specify the reason for restriction (hospital policy, liability concerns, difficulty of the medical record system, medical staff instructions, other). In addition, we asked those who had full access about their documentation skills (patient history, physical examination, medication and investigation orders, and documentation completeness).

Section 2 asked about medical record access experience and patient relationships while using medical records, including finding medical records, finding medical information, asking more questions, maintaining eye contact with the patient, time spent with the patient, and doctor–patient communication. The responses used a 5-point Likert scale from “strongly agree” to “strongly disagree.”

Section 3 asked about the medical students’ choice between paper medical records and EHRs with regard to taking the patients’ medical histories, performing physical examination, entering orders, accessing clinical guidelines, and organizing information. At the end of this section we asked the participants to explain why they preferred the selected type of medical record in an open-ended question format. In addition, there were two extra open-ended questions about any further educational impact of medical records and the medical students’ experience in other hospitals if they worked in more than one.

The reliability of instrument was calculated based on Cronbach’s alpha. The alpha values were measured for full access items, medical record access experience items, and preferences items. The value achieved were .676, .558, and .834, respectively, indicating a satisfactory level of reliability [33].

### Data analyses

Data were analyzed using SPSS Statistics v.19 (IBM Corporation, Armonk, NY, USA). Descriptive statistics for demographic data, level of access, experience, and preference was calculated. The experience with accessing medical records which differed depending on the type of medical record was presented through calculating median and interquartile range (IQR). 5-point Likert scale questionsُُُ' responses were combined into three categories: disagree (1) (combining “strongly disagree” and “disagree”), neutral (2), and agree (3) (combining “strongly agree” and “agree”). Open-ended questions were analyzed using thematic analysis including reviewing and extracting themes, as suggested by Popping [34]. To compare students’ experiences between paper medical records and EHRs, Mann-Whitney U and Kruskal-Wallis tests were performed. Significance was assessed at the 0.05 level.

To address non-response bias, several methods were recommended such as comparing results with population’s characteristics and wave analysis. The second method was selected due to availability of survey data. It involves comparing results of main study’s variable between early responders and late responders. Early responders are considered as responders and late responders are similar to non-responders [35, 36]. The mean response of access experience items between these two groups were compared. The analysis suggested that there is no significant differences between the early responders and late responders at the 0.05 level. Accordingly, there are no significant differences between responders and non-responders. This result increases the confidence that the sample group is representative to the population group.

## Results

**Fig. 1:**
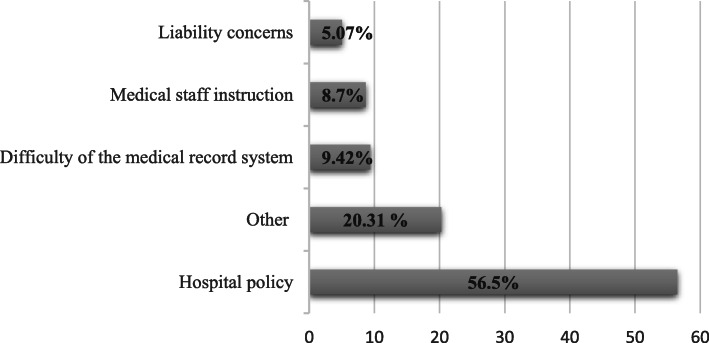
Reasons for restricting participants’ access to medical records

The total number of students who received the email is 2400 students and 388 responded, which resulted in 16.1% response rate. Of the 388 participants, 17 were excluded since they did not practice in hospitals. Therefore, 371 completed surveys were included in the study. Table 1 shows their characteristics. The majority of participants were female (68.5%) aged 18–23 years (71.4%). In addition, 103 (27.8%) of participants were in the fourth year, with 246 (66.3%) of them training in University-owned hospitals; 138 (37.2%) did not have access to medical records; and 56.5% of the 138 participants were restricted by hospital policy (Fig. 1).

**Table 1:** Characteristics of participants

Characteristics	***n*** (371)	(%)
**Gender**		
Male	117	31.5
Female	254	68.5
**Age**		
18–23	265	71.4
24–28	96	25.9
> 28	10	2.7
**Year**		
Second-year	31	8.4
Third-year	46	12.4
Fourth year	103	27.8
Fifth year	43	11.6
Sixth year	76	20.5
Intern	72	19.4
**Type of hospital**		
University-owned hospital	246	66.3
Ministry of Health hospital	82	22.1
Private hospital	5	1.3
National Guard Hospital	12	3.2
Armed Forces Hospital	9	2.4
Security Forces Hospital	2	0.5
Others	15	4.2
**Having access to medical record**		
Yes	233	62.8
No	138	37.2

With regard to access to medical records, 81 (34.8%) of the participants had access through a senior’s account. More than half (66.1%) had access to the EHR system, and the majority (83.27%) had only read-only access (Table 2).

**Table 2:** Access methods, type of medical record, and level of access provided to participants who accessed to medical record

Variable	***n (233)***	%
**Access methods**		
Free to access	67	28.8
Access from IT team	59	25.1
Access through a senior account	81	34.8
Other	26	11.3
**Type of medical record**		
Paper medical record	79	33.9
Electronic health record	154	66.1
**level of access**		
Read- only access	194	83.27
Full access (read, review and enter order/data)	39	16.73

The experience with accessing medical records differed depending on the type of medical record (Table 3). Median was used to report participants’ experiences. Median of all experience items were 3 among paper group. They mostly agree on reaching medical records without effort (67.1%) followed by satisfaction with the doctor–patient communication while using medical records (51.9%). Median of all items were 3 among EHR group expect for eye contact time with patients and spending time with patient, which were 2. EHR group mostly liked reaching medical records easily (70.1%) and mostly disagree about positive impact of using EHR on the eye contact time with patients (37.0%).

**Table 3:** Participants’ experience with accessing medical records

Experience with medical records	Paper medical record (***n*** = 79)					EHR (***n*** = 154)				
	Disagree ***n*** (%)	n Neutral n (%)	Agree ***n*** (%)	Median	IQR	Disagree n (%)	Neutral n (%)	Agree n (%)	Median	IQR
It was easy to reach medical records	12 (15.2)	14 (17.7)	53 (67.1)	3	1	23 (14.9)	23 (14.9)	108 (70.1)	3	1
It was easy to find essential information (e.g. past medical history and medications)	25 (31.6)	22 (27.8)	32 (40.5)	3	2	25 (16.2)	27 (17.5)	102 (66.2)	3	1
The items of medical records encouraged me to ask more history/physical examination questions	9 (11.4)	30 (38.0)	40 (50.6)	3	1	19 (12.3)	37 (24.0)	98 (63.6)	3	1
Using medical records (read /data entry) affected positively on the eye contact time with patients	15 (19.0)	37 (46.8)	27 (34.2)	3	1	57 (37.0)	44 (28.6)	53 (34.4)	2	2
Using medical records (read/data entry) affected positively on the time that should be spent with patients	25 (31.6)	31 (39.2)	23 (29.1)	3	2	54 (35.1)	38 (24.7)	62 (40.3)	2	2
Overall, I was satisfied with the doctor–patient communication while using medical records	8 (10.1)	30 (38.0)	41 (51.9)	3	1	15 (9.7)	40 (26.0)	99 (64.3)	3	2

*IQR* interquartile range

**Fig. 2:**
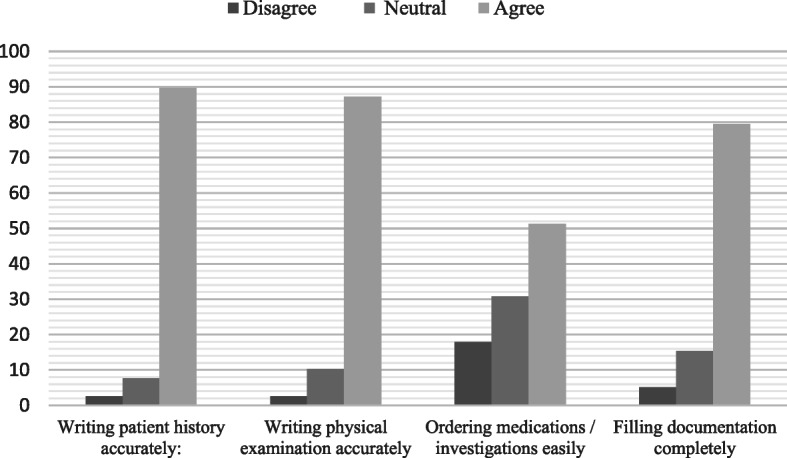
Benefits of full medical record access according to participants

Fig. 2 presents the benefits of providing full medical record access to medical students. Precise writing of patient history was the mostly agreed-on outcome (89.7%), followed by correct writing of physical examination (87.2%), completeness of documentation (79.5%) and ordering (51.3%).

**Fig. 3:**
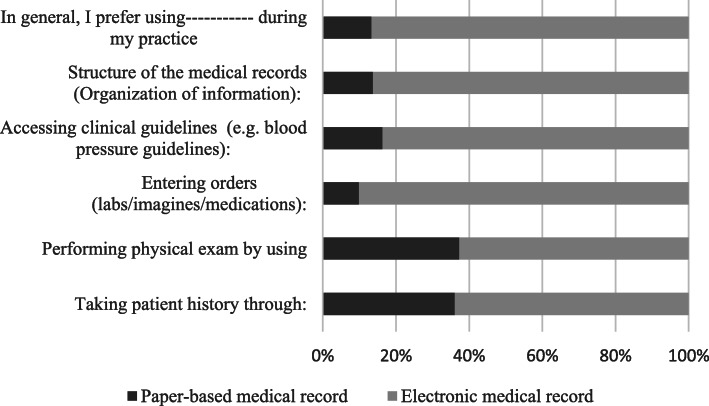
Participants’ preference for the type of medical record they want to use in their future practice

Fig. 3 shows each group’s preference for the type of medical record. The majority (86.7%) would like to use EHRs in their future practice, In addition, 90.1% of participants preferred using EHRs to enter orders, while only 9.9% preferred using paper medical records to enter orders, and 86.3% preferred the organization of information in EHRs. Interestingly, 36.1% and 37.3% of participants liked paper medical records for taking a medical history and performing a physical examination, respectively.

Mann-Whitney U test was performed to test the effects of gender, type of medical record, and level of access on the experience with accessing medical records (Table 4). There was no significant difference in experience between males and females and between participants who had full access compared to those who had read-only access. There was a significant difference between experiences with the type of medical record (*U* = 5200, *P* = .04). The EHR group had a better experience compared to the paper group.

**Table 4:** Effect of participants’ gender, type of medical record, and level of access on their experience with accessing medical records

Characteristics	***n*** (233)	Mean Rank	Sum of Ranks	***Mann-Whitney U***	***P- value***
**Gender**					
Male	75	120.48	9036	5664	.55
Female	158	115.35	18,225		
**Type of record**					
Paper medical record	79	105.82	8360	5200	.04
Electronic medical record	154	122.73	18,901		
**Level of access**					
Read-only only access	194	116.18	22,539.50	3624.50	.65
Full access	39	121.06	4721.50		

Kruskal-Wallis test compared the effect of age, type of hospital, and studying year on the participants’ experience with using medical records (Table 5). There were statistically significant differences in experience with using medical records based on the type of hospital (*x*^2^ = 12.684 , *P* = .048). To know which of the specific groups differed, Mann-Whitney test was performed. Results showed significant differences at the.05 between participants in University-owned hospitals and Ministry of Health hospitals to University-owned hospitals (Mean Rank =122.35). Also, Significant differences between Ministry of Health hospitals and National Guard Hospitals to National Guard Hospital (Mean Rank =147.90).

**Table 5:** Effect of participants’ age, type of hospital, and studying year on their experience with accessing medical records

Characteristics	n (233)	Mean Rank	***x***^**2**^	***P- value***
**Age**				
18–23	139	115.90	2.50	.286
24–28	84	122		
> 28	10	90.30		
**Type of hospital**				
University-owned hospital	142	122.35	12.68	.048
Ministry of Health hospital	57	98.48		
Private hospital	5	76.00		
National Guard Hospital	10	147.90		
Armed Forces Hospital	7	118.79		
Security Forces Hospital	2	169.00		
Others	10	124.60		
**Studying year**				
Second-year	8	50.06	11.01	.051
Third-year	9	110.78		
Fourth year	50	123.69		
Fifth year	33	112.09		
Sixth year	68	122.49		
Intern	65	117.71		

*x*^2^ (Chi-Square)

### Open-ended questions

The survey included three open-ended questions. In regard to reason of preference, most of the responses were from participants who preferred EHRs, and only a few answers were from participants who preferred paper medical records. In addition, one participant liked to organize ideas on paper. The participants stated several benefits of EHRs, and five categories emerged:

- Category 1: legibility and clarity.
- Category 2: accessibility and availability,
- Category 3: data entry and organization,
- Category 4: safety and privacy.
- Category 5: secondary uses of EHR data, such as medical research.

In reference to working in different hospitals, the majority described an unsatisfactory experience with paper medical records because of difficulty in reading and finding information, incompleteness, and disorganization. In contrast, many participants were satisfied with using EHRs, including access and a comprehensive view of patient data. However, a few were disappointed with EHRs because of difficulty in learning, slowness, a lack of features, and inappropriate access. In addition, few participants stated they had a good experience with paper medical records related to easy information access.

Many participants asked for access to medical records and to be trained on how to review and write in medical records before internship. Several participants wanted to acquire skills related to writing in medical records, even as a mock-up model or under their seniors’ supervision, which would help them in their future practice. With regard to educational benefits of medical records, many participants clarified the role of EHRs in directing medical students’ history taking and physical examination as EHRs include all essential information. One participant also suggested considering long cases from medical records as teaching materials that could be incorporated into the medical curriculum.

## Discussion

Medical students in Saudi Arabia require competencies in using medical informatics applications and medical information documentation before graduation [21]. To our knowledge, no studies have investigated medical students accessing medical records in Saudi Arabia that involves student perspectives, different academic institutions and comparing learning experience between EHR and paper medical records. This descriptive study examined medical students’ access to paper medical records and EHRs in Saudi Arabia and compared the experience of accessing paper medical records and EHR from the medical students’ perspective.

Most medical students had access to medical records, which is consistent with the results of Welcher et al. [6], who reported that 96% of medical schools allow students to access medical records. Not surprisingly, most of the medical students in this study accessed EHRs compared to paper medical records, which is similar to other studies [27, 37]. This is an indication of minimal use of paper medical records in Saudi Arabia’s hospitals.

One of the factors that effects on students’ experience is the type of institution. The University owned and National Guard hospitals provide a better experience for medical students. The reason maight be that these hospitals give medical students more opportunities to interact with patient and medical records compared to other types of hospitals. In fact, most of University-owned hospitals provided full access to medical students during 2013–2014 [6].

In this study, hospital policies are the biggest reason for restricting medical students’ access to medical records, which is consistent with the results of Wittels et al. [38]. Having hospital policies as a main barrier for accessing medical record may indicate hospitals concerns related to liability. Liability concerns could raise if students have ability to document in EHR, which is reported as second barrier [38]. A possible solution of this issue is providing feedback to medical students after documentation which enhance documentation skills [38]. This finding also explained why most medical students who have access to medical records use seniors’ accounts, which also mentioned by other study [39]. Beside liability concerns, other studies mentioned other barriers such as billing, reduced productivity, financial concerns, and logistical and structural problems [3, 6, 18]. Regarding financial barrier, the annual license fee ranges from $800 to $3200 per provider. This cost did not include maintenance, hardware, and productivity loss fees [40].

Providing full access of medical record to medical students is necessary as appropriately using EHRs is an important competency that affects patient care and safety [31], as well as increasing EHR familiarity will enhance positive impact resulted from using EHR [11, 12]. Most education deans have reported that medical students’ education would be undesirably affected without involving them in documentation [29]. In fact, many participants stated the importance of providing EHR access to medical students and recommend methods for such access in the open-ended questions.

This study confirmed that medical students who access EHRs had a better experience than those who access paper medical records. Furthermore, most medical students are satisfied with reaching medical records easily and finding essential information while using EHRs. With regard to preference, most of the participants in this study liked the organization of information and access to medical guidelines in EHRs. One study found that most medical students enjoy the ability of EHRs to organize information [28]. In addition, EHRs have the advantage of promoting medical students to ask more questions related to medical history [28], which was also confirmed in this study. However, integrating EHR with medical education led to several disadvantages that may influence on educational outcomes. Using of EHR template and “copy and paste” feature may affect negatively on students’ critical thinking and synthesizing information. Patient – doctor communication may also negatively affect with using EHR [18, 28]. Although, most students prefer using EHRs in their future practice, the main question remains whether paper record offer students all the clinical educational benefits that could be acquired from EHR. In fact, students who accessed paper medical record mostly liked ability to access medical record effortlessly and ability to write more orders [5]. However, current transformation to EHR requires additional skills that not required from students when using traditional paper records like electronic ordering and using of CDSS [7, 8]. Such question needs to be discussed extensively in literature.

Although the SaudiMED framework requires medical students to acquire writing skills and use informatics systems effectively before graduation [21], the majority of participants had read-only access in this study. However, other studies that found that most medical schools allow medical students to write on patients’ records [6, 27, 37]. In fact, in this study, participants who had full access (read, review, and enter order/data) mostly agreed on the educational benefits of medical records, such as accurate writing of a patient’s medical history and physical examination. Therefore, medical students who do not have such experience might miss acquiring and practicing some of these fundamental skills which required by medical graduate students [20, 21].

To ensure proper access of EHRs to medical students, several regulations and hospital policies need to be implemented and proper EHR training incorporated into the medical curriculum. In fact, several organizations recommended principles to support such educational opportunities [4, 19, 41]. In this study, although the participants had access to medical records, their responses to the open-ended questions revealed the necessity of proper control of such access as access accounts had been freely distributed among them and most had access through their seniors’ accounts. Medical students should be provided proper EHR training before proving them access to medical records as it will help them practice and use EHR systems easily. In this study, many participants also disliked the use of the EHR system because of the difficulty of learning and slowness, which may increase the resistance to EHR and minimize the value of using EHRs in their future practice. King Saud University provide such training for third year medical students as a part of medical informatics course.

This study had several limitations. First, it described medical students’ experiences by using a survey. Experiences might vary depending on the type of system used and training hospitals. The actual usage of EHRs was not measured. However, several studies have used surveys as a tool to understand medical students’ experience with using medical records and their impact on education [18, 20, 42]. Future research may depend on actual user data that can be extracted from EHR systems and reflect actual usage. Second, the survey used was self-developed and was not validated, although it was based on several studies that discussed medical students’ access to medical records [6, 28, 31] and was reviewed by several experts prior to distribution. The small sample size is a limitation since the number of the completed survey was less than the required due to removal of 17 surveys. However, the number of students who returned the survey achieved the required sample size. Another limitation is the low response rate, even though several strategies were used to boost the response rate including sending reminders after 2 weeks, adding the survey link in the email, ensuring anonymity of respondents, and extending survey availability [43]. Indeed, web-based respond rate has lower response rate compared to paper-based survey [43]. Future work can explore if paper medical record provide more educational benefits compared to EHR. In addition, it can discover medical school policies and training related to medical students’ access to medical records in Saudi Arabia.

## Conclusion

Accessing medical records helps medical students acquire several fundamental skills for their future practice. Medical students’ experience with EHRs is better compared to paper medical records. Providing read-only access restricts medical students’ educational experience. Incorporating EHR access into the medical curriculum is essential as it will provide new educational opportunities that were not available before.

## Supplementary Information

**Additional file 1.**

## Data Availability

The analysed data used during the study are available from the corresponding author on reasonable request.

## Abbreviations

EHR: Electronic health record
CDSS: Clinical decision support systems
IT: Information technology
LCME: Liaison Committee on Medical Education
SaudiMED: Saudi Medical Education Directives
PLO: Program learning objective
MOH: Ministry of Health
IQR: Interquartile range
*x*^2^: Chi-Square
