# Supplementary material for: Medical students’ experience with accessing medical records in Saudi Arabia: a descriptive study
Source: BMC Med Educ. 2021 May 12;21:272. doi: 10.1186/s12909-021-02715-7 (PMC8117651; doi:10.1186/s12909-021-02715-7)
Supplement: Supplementary file 1 — Additional file 1. [file 12909_2021_2715_MOESM1_ESM.docx]

**Appendix:** **Survey**

Dear Medical Student:

I request you to complete the following questionnaire regarding your experience with accessing medical records. Please complete this survey only once and only if you are a medical student.

Study objectives:

1. Assess medical students’ access to paper medical records and electronic health records (EHRs) in Saudi Arabia.
2. Compare the educational benefits of paper medical records and EHRs from the medical students’ perspective.

If you agree to participate in this study, please answer the questionnaire as best as you can. It should take no longer than 10 minutes of your time. Your responses are of utmost importance to me. Please do not enter your name or contact details; they will remain confidential. This survey is voluntary, and you may refuse to participate at any time.

Sincerely,

Dr. Jwaher Almulhem

Assistant Professor in Health Informatics

Medical Informatics & eLearning Unit (MIELU)
Medical Education Department
College of Medicine
King Saud University

Inclusion criteria question: Are you a medical student/intern in Saudi Arabia?

- Yes
- No

1. Gender:

- Male
- Female

2. Age (years):

- 18–23
- 24–28
- >28

3. Which year are you studying in?

**Note: The first year is counted as orientation year (سنه تحضيريه).**

- Second year
- Third year
- Fourth year
- Fifth year
- Sixth year
- Intern

**Note: Even if you have worked in more than one hospital, please describe your experience in only one hospital and discuss your experience in others in the last open-ended question.**

1. Which type of hospital have you worked in?
   - University-owned hospital
   - Ministry of Health (MOH) hospital
   - Private hospital
   - National Guard hospital
   - Armed forces hospital
   - Security forces hospital
   - Others (please specify): _____

5. Have you accessed medical records in this hospital (reviewed or entered order/data)?

- Yes
- No

6. If no, please specify the reason and kindly submit the survey:

- - Hospital policy (e.g., hospital policy prevents access to medical records for interns/students)
  - Liability concerns (e.g., concern about incorrect data entry)
  - Difficulty of the medical record system
  - Medical staff instructions (e.g., a senior asked not to access medical records)
  - Other (please specify): _____

**If so, kindly answer the remaining questions.**

1. How did you get access?

- Free to access (paper medical records)
- Access from the information technology (IT) team
- Access through a senior’s account
- Other (please specify): _____

8. What type of medical record have you used in this hospital?

- Paper medical record
- EHR

9. What level of access have you been provided?

- Read-only access
- Full access (read, review, and enter order/data)

10. If you have full access to medical records**, kindly answer the following questions:**

| After using the selected type of medical record: | Strongly agree | Agree | Neutral | Disagree | Strongly disagree |
| --- | --- | --- | --- | --- | --- |
| - I was able to write the patient’s medical history accurately. |  |  |  |  |  |
| - I was able to write the patient’s physical examination accurately. |  |  |  |  |  |
| - I was able to order medications/investigations easily. |  |  |  |  |  |
| - Overall, I was able to fill in documentation completely. |  |  |  |  |  |

11. On the basis of the selected type of medical record, please choose a suitable answer that describes your experience with medical records and patient relationship.

| Experience with the medical record: | Strongly agree | Agree | Neutral | Disagree | Strongly disagree |
| --- | --- | --- | --- | --- | --- |
| - It was easy to reach medical records. |  |  |  |  |  |
| - It was easy to find essential information (e.g., past medical history and medications). |  |  |  |  |  |
| - The items of medical records encouraged me to ask more history/physical examination questions. |  |  |  |  |  |
| - Using medical records (read/data entry) affected negatively the eye contact time with patients. |  |  |  |  |  |
| - Using medical records (read/data entry) affected negatively the time that should be spent with patients. |  |  |  |  |  |
| - Overall, I was satisfied with doctor–patient communication while using medical records. |  |  |  |  |  |

12. Depending on your experience with the medical records, what do you prefer?

| **Items** | Paper medical record | EHR |
| --- | --- | --- |
| - Taking patient history through: |  |  |
| - Performing physical examination using: |  |  |
| - Entering orders (labs/imagines/medications): |  |  |
| - Accessing clinical guidelines (e.g., blood pressure guidelines): |  |  |
| - Structure of the medical records (organization of information): |  |  |

13. In general, I prefer using _____ during my practice.

- paper medical records
- EHRs

**Please indicate the reasons:**

14. Describe your experience with medical records in other hospitals (if you have worked there).

15. Kindly give us your comments on any further educational impact of medical records.

Thank you for your time
